# The Role of AMPKα in the Mechanism of Development and Treatment of Heart Failure

**DOI:** 10.31083/RCM36391

**Published:** 2025-08-30

**Authors:** Yue Feng, Zixiong Zhu, Yubin He, Xuewen Li

**Affiliations:** ^1^Department of Cardiovascular Medicine, Third Hospital of Shanxi Medical University, Shanxi Bethune Hospital, Shanxi Academy of Medical Sciences, 030032 Taiyuan, Shanxi, China

**Keywords:** AMPK*α*, heart failure, energy metabolism, mitochondrial dysfunction, autophagy, oxidative stress, AMPK agonists

## Abstract

The AMP-activated protein kinase (AMPK) alpha (AMPK*α*) subunit is the catalytic subunit in the AMPK complex and includes both *α*1 and *α*2 isoforms. Phosphorylation of upstream kinases at the Thr172 site in the *α*-subunit is critical for AMPK activation. The kinases upstream of AMPK include liver kinase B1 (LKB1), calcium/calmodulin-dependent protein kinase kinase *β* (CaMKK*β*), and transforming growth factor *β*-activated kinase 1 (TAK1). LKB1 predominantly regulates the AMPK*α*2 isoforms, whereas the phosphorylating roles of CaMKK and TAK1 in different isoforms of AMPK*α* have yet to be properly defined. Moreover, the understanding of the roles of AMPK*α*1 and *α*2 remains limited. Significant differences exist between the AMPK*α*1 and AMPK*α*2 isoforms regarding tissue distribution, cellular localization, and cardiac-unique roles, with AMPK*α*2 being the predominant catalytic isoform in the heart. During heart failure (HF), activated AMPK*α* isoforms, particularly AMPK*α*2, promote the remodeling of energy metabolism, ameliorate mitochondrial dysfunction, activate mitophagy, attenuate oxidative stress, and reduce cardiomyocyte death, thereby protecting cardiac function and delaying HF progression. Thus, drugs that selectively activate AMPK complexes containing specific *α*2 isoforms may help treat HF. However, AMPK activators are not currently very subtype-selective, direct agonists remain in clinical trials, and indirect agonists, although widely used in the clinic, have some non-AMPK-dependent effects. Therefore, a compelling need exists to develop subtype-selective activator drugs with greater specificity and efficacy and fewer side effects.

## 1. Introduction

Heart failure (HF) is a complex clinical syndrome characterized by high morbidity, hospitalization, and mortality as a result of the advanced progression of multiple cardiac diseases. According to epidemiological data, the prevalence of HF increased by 29% globally between 2010 and 2019, and there are currently approximately 56.2 million HF patients [1]. The main clinical manifestations of HF are dyspnea, activity limitation, and fluid retention—symptoms that severely reduce patient quality of life and life expectancy. However, significant progress has recently been made in the pharmacologic treatment of HF, especially with the introduction of the sodium–glucose cotransporter 2 (SGLT2) inhibitor (SGLT2i), which has transformed the therapeutic regimen of HF from the traditional “Golden Triangle” to the “New Quadruple Therapy”, and has greatly reduced the readmission and mortality rates of HF patients. Nonetheless, the prognosis for HF patients remains poor, with one-year mortality rates ranging from 25% to 75% [2]. Therefore, detailed exploration of the molecular mechanisms involved in HF and searching for new therapeutic targets are essential for improving patient prognoses.

AMP-activated protein kinase (AMPK) is a key serine/threonine protein kinase, whose activity was studied from the early 1970s until 1994, when it was identified as a peripheral “energy sensor” under conditions of energy deprivation, ischemic stress, and strenuous exercise [3]. AMPK is usually inactive under normal physiological conditions; however, AMPK is activated under stress conditions. Activated AMPK restores energy homeostasis by promoting the catabolic ATP production pathway and inhibiting the anabolic energy expenditure pathway. AMPK activation depends on the phosphorylation of the α subunit at the Thr172 site by upstream kinases. Activated AMPK alpha (AMPKα) has been shown to improve substrate metabolism and energy supply in the heart, thereby slowing the progression of HF. Additionally, AMPKα is involved in regulating critical processes such as mitochondrial dysfunction, autophagy, oxidative stress, and cell death during the development of HF and plays an important role in ameliorating HF. AMPKα also has a subtype-specific role in the pathogenesis of HF. Therefore, this article reviews the links between AMPKα and HF to provide a reference for experimental studies and clinical treatment of HF.

This article also reviews the basic structure of AMPK, its mechanism of activation, and the differences between AMPKα isoforms. The regulatory mechanisms of AMPKα in HF are also summarized. These include energy metabolism, mitochondrial dysfunction, autophagy, oxidative stress, and cell death. Finally, commonly available AMPK activators are described, and their use in treating HF is discussed. Given the subtype-specific role of AMPKα in the onset and progression of HF, developing drugs that target and activate specific α subtypes in the AMPK complex may be important for the clinical treatment of HF.

## 2. General Structure of AMPK

**Fig. 1.**
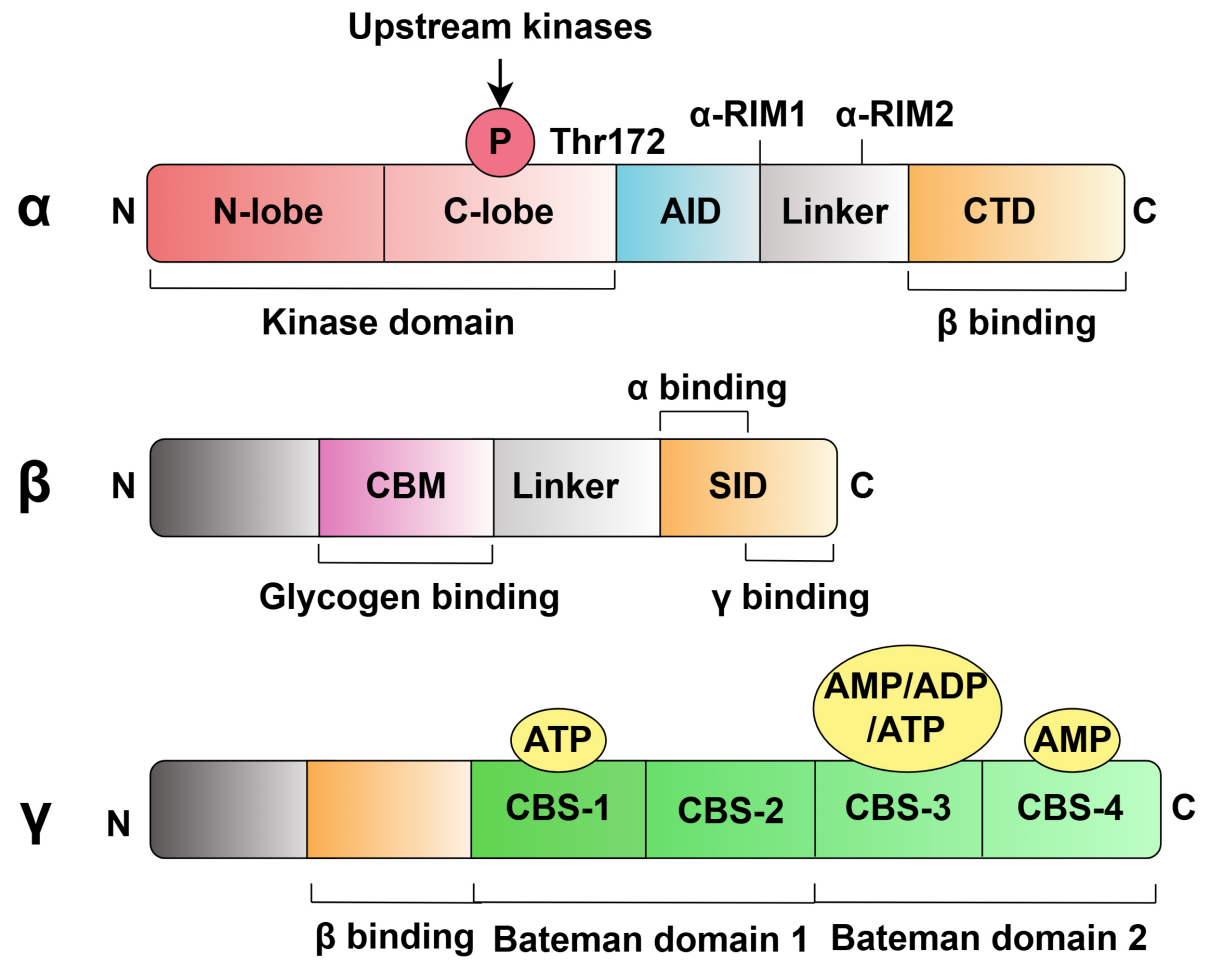
**The mammalian AMPK complex structure**. The AMP-activated protein kinase (AMPK) is a heterotrimeric protein complex containing a catalytic subunit (α subunit) and two regulatory subunits (β and γ subunits). The structural domains in the α-subunit, in order from N-terminal to C-terminal, are the kinase domain (KD), the autoinhibitory domain (AID), the linker region containing two regulatory-subunit-interacting motifs (α-RIM), and the C-terminal domain (CTD), which binds to the β-subunit. When the upstream kinase phosphorylates the threonine residue Thr172 in the KD, the AID-mediated inhibition of the KD is abolished, and AMPK is activated. The β subunit contains a carbohydrate-binding module (CBM), a linker region, and a C-terminal subunit interaction domain (SID) responsible for interacting with the α*–γ* subunits. Meanwhile, excessive binding of glycogen to CBM reduces AMPK activity. The γ subunit contains a short sequence that binds to the β subunit and four tandemly repeated cystathionine-β-synthase (CBS) motifs, forming two Bateman domains. CBS-1 and CBS-4 can be occupied by ATP and AMP, respectively, and CBS-2 cannot bind nucleotides, whereas CBS-3 competitively binds nucleotides (AMP, ADP, or ATP) and thus participates in the allosteric activation of AMPK. AMP, adenosine monophosphate; ADP, adenosine diphosphate; ATP, adenosine triphosphate.

AMPK is a highly evolutionarily conserved heterotrimeric complex with a catalytic α subunit and two regulatory β and γ subunits. The α subunit exists in two isoforms (α1, α2), the βsubunit in two isoforms (β1, β2), and the γ subunit in three isoforms. Theoretically, 12 different AMPK complexes can be formed from various combinations of these subunits [4]. The specific structures of these AMPK complexes are closely related to the regulation of their activity. As shown in Fig. 1, the α subunit contains a kinase domain (KD) and an autoinhibitory domain (AID). When a higher-order kinase phosphorylates the threonine residue Thr172 in the KD, the inhibitory effect of the AID on the KD is abolished, thereby activating AMPK [5]. The β subunit contains a carbohydrate-binding module (CBM) that binds AMPK to glycogen. Excessive accumulation of glycogen binding to CBM may inhibit AMPK activity; however, the exact molecular mechanism has yet to be fully elucidated [6]. Additionally, there is an altered drug and metabolite binding site (ADaM) between CBM and the KD in the α subunit, the major region where exogenous small-molecule activators bind to AMPK. The γ subunit contains four tandem cystathionine β-synthase (CBS) motifs: CBS-1 to CBS-4. Notably, CBS-1 and CBS-4 are occupied by adenosine triphosphate (ATP) and adenosine monophosphate (AMP), respectively, while CBS-3 competitively binds nucleotides (AMP, adenosine diphosphate (ADP), or ATP), while CBS-2 does not [7, 8]. The binding of AMP/ADP to CBS-3 causes the AMPK complex to undergo a significant conformational change, which allows CBS-2 and CBS-3 to bind to the α-regulatory-subunit-interacting motif 1 (α-RIM1) and α-regulatory-subunit-interacting motif 2 (α-RIM2) in the α subunit, respectively, which leads to dissociation of the AID from the KD, promoting phosphorylation and inhibiting dephosphorylation of Thr172, thereby facilitating the allosteric activation of AMPK [9]. In general, the α, β, and γ subunits are involved in regulating AMPK activity, with the α subunit playing a more critical role in AMPK activation.

## 3. Kinases Upstream of AMPK

The mechanism involved in AMPK activation generally encompasses the allosteric regulation of the γ subunit by AMP, the regulation of Thr172 phosphorylation by upstream kinases, and the regulation of Thr172 dephosphorylation by protein phosphatases. Each activation pathway depends on modifying the phosphorylation/dephosphorylation Thr172 phosphorylation site in the KD of the α subunit. In particular, Thr172 corresponds to threonine 174 in AMPKα1 and threonine 172 in AMPKα2 [10]. Three AMPK kinases are present in the heart, including liver kinase B1 (LKB1), calcium/calmodulin-dependent protein kinase kinase β (CaMKKβ), and transforming growth factor β-activated kinase 1 (TAK1), all of which activate AMPK through direct phosphorylation of the Thr172 site [11]. LKB1 is the main upstream kinase that phosphorylates the Thr172 site in the heart and can phosphorylate the Thr172 site in both an AMP-dependent and AMP-independent manner. Blocking LKB1 in mice significantly reduced the level of AMPKα2 activation, whereas the level of AMPKα1 activation was only slightly reduced [12]. This suggests that the regulation of AMPKα1 activity in cardiomyocytes differs from that of AMPKα2 and is not entirely dependent on LKB1 expression. CaMKKβ, which is expressed at lower levels in cardiomyocytes compared to LKB1, provides an alternative activation pathway that is not dependent on LKB1 and AMP and activates AMPKα in response to an increased intracellular Ca^2+^ concentration [13]. TAK1 is a key regulator of both cardiomyocyte survival and death. Although the exact mechanism through which TAK1 activates AMPKα has yet to be fully elucidated, it is known that deletion of both LKB1 and CaMKKβ does not affect TAK1-mediated AMPKα activation [14, 15]. The selective activation of the AMPKα1 and α2 subtypes in the heart by CaMKKβ and TAK1 remains unclear and requires further study.

## 4. Tissue Distribution, Cellular Localization, and Distinct Role of AMPKα in Heart Failure

AMPKα1 and α2 are two isoforms of the α subunit, and comprise 548 amino acids (63 kDa) encoded by the *PRKAA1* gene and 552 amino acids (63 kDa) encoded by the *PRKAA2* gene, respectively [16]. The AMPKα1 and α2 isoforms differ significantly in tissue distribution, cellular localization, and distinct roles in the heart. AMPKα1 is expressed in many tissues and organs, while AMPKα2 is expressed at higher levels in the heart, liver, and skeletal muscle [17]. In the cardiovascular system, AMPKα1 is mainly found in endothelial cells, vascular smooth muscle cells, and fibroblasts, while AMPKα2 is primarily expressed in cardiomyocytes [18]. Moreover, AMPKα1 is mainly localized in the cytoplasm, whereas AMPKα2 is mainly localized in the nucleus [19]. The study conducted using AMPKα1 and α2 knockout or overexpression mouse models further revealed the different roles of these two isoforms in the heart. In a normal heart, AMPKα2 plays a dominant role. Under physiological conditions, baseline cardiac function and cardiac size were not altered in AMPKα1 and α2 knockout mice; however, changes in cardiac mitochondrial function in these mice, such as reduced complex I substrate respiration, reduced complex I and IV activities and altered mitochondrial cristae morphology, resulting in the inability of these mice to increase cardiac output in proportion to increased exercise loads, which manifested as reduced exercise resistance [20]. AMPKα2 knockout mice did not exhibit cardiac atrophy or hypertrophy; a reduced respiratory complex function was observed, similar to AMPKα1 and α2 double knockout mice [21]. This shows that AMPKα subunits play an important role in cardiac mitochondrial function, with AMPKα2 likely being the predominantly functioning isoform. AMPKα2 expression was reduced in failing hearts, whereas AMPKα1 expression was increased [22]. In AMPKα2 knockout mice, pressure overload-induced left ventricular hypertrophy and dysfunction were exacerbated, and the consequent upregulation of AMPKα1 did not fully compensate for the impairment of cardiac function caused by AMPKα2 deletion; meanwhile, AMPKα2 overexpression prevented the development of pressure overload-induced HF [23, 24]. Alternatively, the specific deletion of AMPKα1 had no adverse effect on pressure overload-induced heart function in mice, but AMPKα1 overexpression specifically activated the protein kinase Cζ/activating protein-1 (AP-1) signaling pathway [25]. Although AP-1 activation by AMPKα1 alone cannot affect cardiac function or hypertrophy, AP-1 may play a deleterious role in HF through multiple pathways. These findings suggest that AMPKα2 has a protective effect in both normal and failing hearts, and the development of drugs that can alter or block the reduced expression of AMPKα2 in the failing heart could lead to a breakthrough in HF therapy.

## 5. Regulation of AMPKα in Heart Failure

**Fig. 2.**
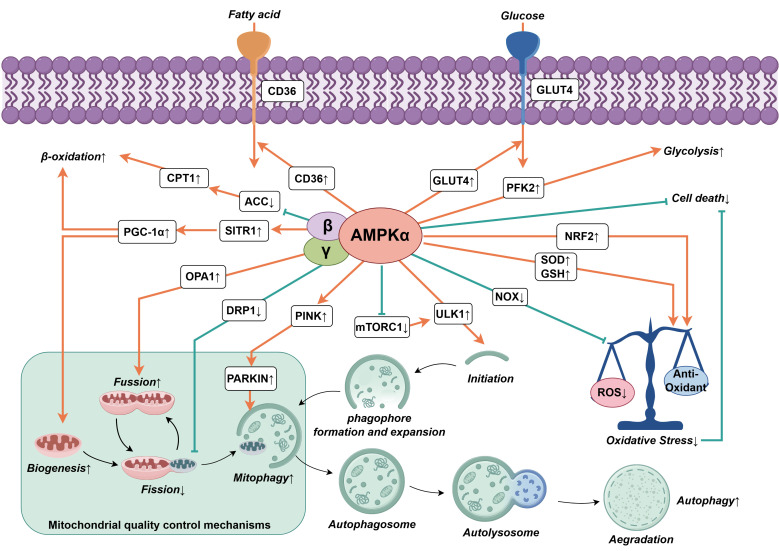
**Role of AMPKα in the regulation of physiological processes associated with heart failure**. As a key regulator of cellular energy homeostasis, AMPKα affects pathways of myocardial energy metabolism and positively influences cardiac function through various mechanisms, such as improving mitochondrial dysfunction, promoting autophagy, inhibiting oxidative stress, and reducing cell death. Black upward arrows (↑) indicate activation, while black downward arrows (↓) represent inhibition. Abbreviations: CD36, fatty acid transporter; ACC, acetyl-CoA carboxylase; CPT1, carnitine palmitoyl transferase 1; SIRT1, silent information regulator sirtuin 1; PGC-1α, peroxisome proliferator-activated receptor gamma coactivator 1α; OPA1, optic atrophy protein 1; DRP1, intracytoplasmic dynamin-related protein 1; PINK, PTEN-induced putative kinase 1; PARKIN, E3 ubiquitin-protein ligase Parkin; mTORC1, mechanistic target of rapamycin complex 1; ULK1, unc-51-like autophagy activating kinase 1; ROS, reactive oxygen species; NOX, nicotinamide adenine dinucleotide phosphate oxidase; SOD, superoxide dismutase; GSH, glutathione peroxidase; NRF2, nuclear factor erythroid 2-related factor 2; PFK2, phosphofructokinase 2; GLUT4, glucose transporter 4.

The AMPKα signaling pathway plays a key regulatory role in the development of HF, involving energy metabolism, mitochondrial dysfunction, autophagy, oxidative stress, and cell death (summarized in Fig. 2). In a state of HF, AMPKα maintains energy metabolism homeostasis by regulating the uptake and utilization of fatty acids (FAs) and glucose. Simultaneously, AMPKα promotes the dynamic balance of mitochondrial biogenesis (MB), fusion, fission, and selective autophagy to improve mitochondrial dysfunction. AMPKα also reduces reactive oxygen species (ROS) generation and enhances antioxidant defenses, alleviating oxidative stress. Additionally, AMPKα effectively restores cardiac function and delays the progression of HF by regulating multiple cell death pathways [26, 27].

### 5.1 Energy Metabolism

As one of the most metabolically demanding organs in the body, the adult heart can utilize a wide range of substrates to produce the ATP required to maintain its function. Typically, more than 60% of the energy is derived from fatty acid (FA) oxidation, with the remainder coming from the metabolism of glucose, lactate, the oxidation of ketone bodies, and particularly, the catabolism of glucose [28]. In the early stages of HF, myocardial substrate utilization is relatively normal, with FA oxidation increasing slightly or remaining constant and glucose utilization increasing [29]. With the progression of HF, the substrate preference of cardiomyocytes gradually shifts to glucose, while FA and glucose oxidation capacity gradually decrease, severely affecting the energy supply to cardiomyocytes [30]. AMPKα improves substrate utilization and energy supply in the failing heart, thus delaying the progression of HF. Activation of the LKB1/AMPKα2 signaling pathway promotes the translocation of CD36 to the plasma membrane, increasing cardiac uptake of long-chain FAs [31]. Additionally, activated AMPKα enhances β-oxidation of FA by affecting acetyl-CoA carboxylase/carnitine palmitoyl transferase 1, peroxisome proliferator-activated receptor α, peroxisome proliferator-activated receptor gamma coactivator 1α (PGC-1α), and other signaling pathways. Enhanced FA uptake and oxidative metabolism help improve the imbalance between energy supply and demand in the failing heart. Simultaneously, AMPKα2 activation promotes translocation of the glucose transporter 4 and stimulates glucose uptake in the heart, which is thought to be a short-term cardioprotective and adaptive response [32]. AMPKα2 also increases glycolysis by regulating phosphofructokinase 2; however, this process may lead to the production of lactate and protons by anaerobic glycolysis, which reduces cardiac contractility and efficiency [33]. Notably, although the functional glycogen-binding domain is located in the AMPKβ subunit, reduced glycogen levels were observed in the cardiac tissues of AMPKα2 knockout mice, suggesting that AMPKα2 may play a role in regulating glycogen levels [34]. Overall, AMPKα is a key regulator of energy metabolism and improves energy metabolism in patients with HF by increasing FA uptake and oxidative metabolism and enhancing glucose transport and glycolysis; AMPKα2 plays a key role in energy metabolism in HF.

### 5.2 Mitochondrial Dysfunction

Mitochondria are key organelles in eukaryotic cells, and their oxidative metabolism produces about 95% of the ATP in the heart. Mitochondria are also involved in cellular activities such as metabolic regulation, signal transduction, cell proliferation, differentiation, and apoptosis. However, mitochondrial dysfunction is prevalent in HF and is an adaptive response to energy metabolism pathway disruptions, but fails to restore energy metabolism effectively and instead exacerbates cardiac dysfunction and myocardial injury [35]. To defend against mitochondrial damage, cardiomyocytes maintain mitochondrial homeostasis through mitochondrial quality control mechanisms such as MB, fusion, fission, and mitophagy. However, MB, and fusion processes are often inhibited, and fission is overactivated in the state of HF. PGC-1α is a key regulator of MB, and its reduced expression in HF models leads to MB inhibition and a decrease in the number of mitochondria [36, 37]. AMPKα either directly phosphorylates PGC-1α or activates silent information regulator sirtuin 1 (SIRT1) to deacetylate PGC-1α, which increases its expression and promotes MB [38]. However, MB remained induced in AMPKα knockout mice, suggesting that AMPKα is not a determinant of this process and that other molecular regulatory mechanisms are involved [39]. Mitochondrial fusion is regulated by optic atrophy protein 1 (OPA1), mitochondrial fusion protein 1 (MFN1), and mitochondrial fusion protein 2 (MFN2). OPA1 expression is generally downregulated in failing hearts, resulting in a decrease in fusion and an increase in small and fragmented mitochondria [40]. The MFN1 and MFN2 levels are unchanged in the failing human heart, although MFN1 phosphorylation at the Ser86 site and inhibition of GTPase activity are detected in the hearts of HF rats [40, 41]. AMPKα promotes OPA1-mediated fusion, inhibits mitochondrial fragmentation, and maintains mitochondrial integrity [42]. Mitochondrial fission is mainly mediated by the intracytoplasmic dynamin-related protein 1 (DRP1) and its receptor mitochondrial fragmentation factor (MFF). Clinical data suggest that serum levels of DRP1 in HF patients are not significantly different from those in healthy controls; however, hyperactivation of DRP1 has been observed in rodent models of HF, increasing fission [43, 44]. AMPKα inhibits fission by decreasing DRP1 phosphorylation at the Ser616 site while increasing phosphorylation at the Ser637 site; meanwhile, AMPKα also simultaneously enhances fission by promoting MFF phosphorylation at the Ser155 and Ser172 sites, upregulating DRP1 expression, and promoting DRP1 mitochondrial translocation [45, 46]. Notably, AMPKα deficiency promotes fission, suggesting a predominantly inhibitory role [47]. In conclusion, AMPKα plays a cardioprotective role in HF by promoting MB and fusion and inhibiting excessive fission. The regulation of mitophagy by AMPKα is described in detail in the following autophagy section. Mitochondrial activity interactions are extremely complex and involve a variety of molecules and processes, such as PGC-1α regulation of MFN1, MFN2, and DRP1, which are involved in fusion and fission, OPA1 cleavage to promote fission, MFN2 involved in mitochondria–endoplasmic reticulum translocation and mitophagy, and DRP1, which promotes mitophagy. Further studies are required to improve understanding of the interplay involved in the mitochondrial quality control mechanisms in HF and the effects of AMPKα on these mechanisms.

### 5.3 Autophagy

Autophagy is a process of either the selective or non-selective removal of damaged organelles and cytoplasmic components, which includes autophagy initiation, isolation membrane (phagophore) formation and expansion, membrane closure to form autophagosomes, fusion of autophagosomes with lysosomes, and degradation of contents. Based on unc-51-like autophagy activating kinase (ULK) status and autophagosomes origin, autophagy can be categorized into microtubule-associated protein 1 light chain 3 (LC3)-dependent classical autophagy and ULK1/Ras-related protein 9 (Rab9)-dependent alternative autophagy [48]. Under normal conditions, autophagy in the heart contributes to cardiomyocyte survival and maintenance of energy metabolism homeostasis. In HF, the mitochondria in cardiomyocytes are impaired, and their dysfunction further exacerbates HF by creating a cycle of damaging disturbances in energy metabolism, oxidative stress, and cell death. Cells can selectively remove damaged mitochondria through an autophagic mechanism known as mitophagy. In the transverse aortic constriction (TAC) mouse model, classical autophagy is activated within one day of a TAC, peaks and then rapidly falls back to baseline levels without involving mitophagy activation; alternative mitophagy is increased three to seven days after TAC but decreases after seven days to become inactivated after fourteen days, leading to mitochondrial dysfunction and HF development [49, 50]. This demonstrates that the changes in mitophagy are closely related to the development of HF and that it is the main form of autophagy that protects cardiac function. AMPK is a potent activator of autophagy and initiates autophagy either by directly or indirectly inhibiting the mechanistic target of rapamycin complex 1 (mTORC1) by phosphorylating the mTORC1 component, Raptor, or the negative regulator, tuberous sclerosis complex 2 [51]. AMPK can also activate autophagy in an mTOR-independent manner, such as by phosphorylating autophagy-associated proteins in ULK1 and the phosphatidylinositol 3-kinase catalytic subunit type 3/vacuolar protein sorting 34 complex, or indirectly by regulating autophagy-associated genes downstream of transcription factors [52]. The recent study has confirmed that AMPKα activation promotes moderate mitophagy and restores mitochondrial function to improve HF. In a mouse model of HF, when HF occurred, AMPKα2 expression and activity were elevated five days after TAC, but decreased fourteen days after TAC [22]. The expression and activity of AMPKα2 were consistent with the level of mitophagy in cardiac tissues of mice with HF, and its downregulation led to impaired mitophagy and exacerbated HF. Further studies have revealed that AMPKα2 overexpression phosphorylates the Ser495 site in PTEN-induced putative kinase 1 (PINK1), activates the PINK1/E3 ubiquitin-protein ligase Parkin/Sequestosome 1 pathway, and increases the level of mitophagy in early HF [22]. Therefore, AMPKα2-mediated mitophagy is closely related to HF, and further studies are needed to clarify the mechanism through which AMPKα2 regulates mitophagy in cardiomyocytes and provide a new target for HF intervention and treatment.

### 5.4 Oxidative Stress

Oxidative stress is a state of redox imbalance due to excessive ROS and an impaired antioxidant defense system. Intracellular ROS are mainly derived from mitochondria, reduced nicotinamide adenine dinucleotide phosphate oxidase (NOX), and lipid oxidase [53]. Under normal physiological conditions, the amount of intracellularly produced ROS is low, involved in cell signaling and functional regulation, and can be scavenged by the antioxidant system to maintain redox balance [54]. However, in HF, mitochondrial dysfunction significantly increases ROS levels beyond the scavenging capacity of the antioxidant system, leading to ROS accumulation. Excess ROS accumulation causes oxidative damage to mitochondrial components, creating a damaging cycle leading to cardiomyocyte calcium overload, apoptosis, inflammatory damage, and myocardial fibrosis, thus exacerbating HF [55, 56]. Several studies have confirmed that AMPKα reduces ROS levels in HF [26, 57]. For example, AMPKα inhibits NOX-mediated ROS production, promotes uncoupling protein 2 expression to reduce ROS production, attenuates the reduction in the activity of cardiac antioxidant enzymes and promotes nuclear transcription factor red lineage 2-associated factor 2 (NRF2), a major regulator of the antioxidant defense system, regarding its activity and expression [26, 57]. Notably, NOX4 expression was significantly increased during HF, while AMPKα inhibited NOX4 expression and attenuated oxidative stress and cell death in cardiomyocytes [58, 59]. Additionally, AMPKα1 directly phosphorylates the Ser374, 408, and 433 sites in NRF2 and regulates the activation of NRF2 downstream molecules [60]. AMPKα1 activation promotes NRF2 activity, thereby attenuating ischemia-reperfusion (I/R)-induced cardiac dysfunction, apoptosis, and myocardial fibrosis [61]. Activated AMPKα2 also attenuates pressure overload-induced HF by promoting NRF2/heme oxygenase-1 signaling, inhibiting NOX activity and restoring superoxide dismutase (SOD) activity to regulate oxidative stress [62]. Taken together, AMPKα attenuates oxidative damage in HF by decreasing ROS production and increasing ROS clearance. Notably, ROS can also regulate the activity of AMPKα, forming a complex regulatory loop [63]. Future studies should further elucidate the relationship between AMPKα and ROS in cardiomyocytes, and based on this, develop novel therapeutic strategies targeting ROS and their regulatory networks to provide new directions for the prevention and treatment of HF.

### 5.5 Cell Death

Cell death is when a cell loses its viability and vital functions under specific conditions and eventually fails to maintain normal metabolism and life activities. Based on morphological features, cell death can be categorized into apoptosis, necrosis, autophagic cell death, and mitotic catastrophe. Since mammalian cardiomyocytes exit the cell cycle shortly after birth and their ability to return to the cell cycle is controversial, mitotic catastrophe does not represent a common form of cardiomyocyte death [64]. Many new forms of cell death have also been discovered following increasingly detailed study on cell death mechanisms, such as necroptosis, ferroptosis, and pyroptosis, all of which have unique mechanisms and biological significance. The loss of cardiomyocytes plays an important role in HF pathogenesis, and progressive loss of cardiomyocytes due to multiple forms of cell death is a key factor. AMPKα has been shown to alleviate HF by regulating various forms of cell death within cardiomyocytes, reducing cardiomyocyte loss, and restoring cardiac contractility. In a DOX-induced HF model, activated AMPKα inhibits ROS production and attenuates oxidative stress-induced cardiomyocyte apoptosis by increasing uncoupling protein 2 expression, an effect that can be abrogated by *AMPKα2* gene silencing [26]. Moderate enhancement of autophagy during early-stage HF promotes cardiomyocyte survival, whereas excessive autophagy activation in late-stage HF induces cardiomyocyte death. Given that AMPKα promotes autophagy, inhibition of the AMPKα/mTOR pathway in HF mice reduced excessive autophagy and alleviated cardiomyocyte apoptosis, ultimately improving cardiac function [27]. Beyond apoptosis and autophagy, AMPKα regulates other programmed cell death forms in cardiomyocytes. The inhibitory effect of AMPKα on ferroptosis is mediated through its regulation of oxidative stress. Specifically, AMPKα attenuates oxidative stress in I/R-induced rat cardiac tissues by downregulating NOX4 and increasing NRF2 expression, thereby mitigating ferroptosis and mitochondrial damage [59, 65]. Importantly, AMPKα2 was pivotal in counteracting I/R-induced ferroptosis [66]. Furthermore, AMPKα exerts anti-oxidant and anti-pyroptosis effects by inhibiting NLRP3 inflammasome activation and caspase-1 cleavage, protecting cardiomyocytes against high glucose-induced pyroptosis [67]. In conclusion, AMPKα plays a role in all forms of cell death in cardiomyocytes, and further investigation into the regulatory mechanisms of AMPKα on different forms of cell death, as well as the interactions of various forms of cell death, will provide new potential therapeutic targets for treating HF.

## 6. AMPK Agonists

AMPK agonists can be classified as direct or indirect based on their activation mechanisms. Direct agonists bind to specific AMPK subunit binding sites, including the α subunit, the ADaM site, and the γ subunit, thereby directly promoting AMPK activation. Indirect agonists do not interact with AMPK but enhance its activity through other pathways. Currently, most indirect agonists are considered to activate AMPK by elevating the intracellular AMP:ATP ratio. The following sections discuss representative drugs from both categories and their therapeutic potential in HF, including cases where the AMPKα signaling pathway is potentially involved (summarized in Table 1, Ref. [24, 68, 69, 70, 71, 72, 73, 74, 75, 76, 77, 78, 79, 80, 81, 82, 83, 84, 85, 86, 87, 88, 89, 90, 91]).

**Table 1.** **Pharmacological effects of different AMPK activators and their use in heart failure**.

Compound	Structure	AMPKα Isoform	Mechanism of AMPK activation	Downstream effect	Refs
PT-1	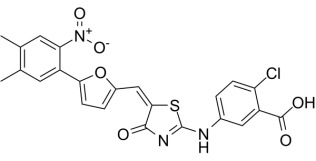	α1	Binded to α subunit	Increased autophagy	[68, 69]
C24	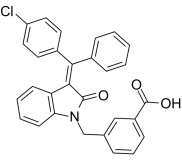	uncertain	Binded to α subunit	No report of cardioprotection	[70]
ZLN024	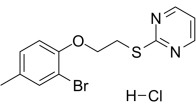	α2>α1	Binded to α subunit	No report of cardioprotection	[71]
A-769662	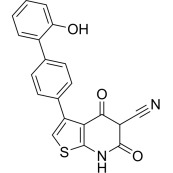	α2>α1	Binded to the ADaM site	Increased systolic function of the heart and decreased apoptosis	[72, 73, 74, 75]
AICAr	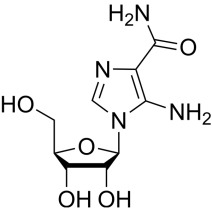	α2>α1	Binded to adenylate binding sites on γ subunits	Inhibited ferroptosis, decreased apoptosis, and maintained autophagy homeostasis	[76, 77, 78, 79, 80, 81, 82]
Metformin	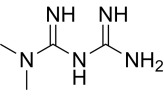	uncertain	Inhibited complex I	Improved mitochondrial respiration; Increased autophagy	[24, 83, 84]
Canagliflozin	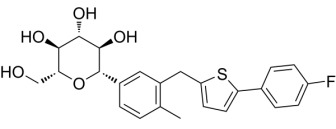	uncertain	Inhibited complex I	Reduced mitochondrial oxidative stress	[85, 86, 87]
Resveratrol	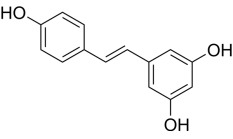	uncertain	Inhibited ATP synthase and phosphodiesterase	Activation of AMPK increased autophagy; Inhibition of AMPK decreased autophagy; Inhibited ferroptosis	[88, 89, 90, 91]

ADaM, altered drug and metabolite binding site; AICAr, 5-aminoimidazole-4-carboxamide ribonucleoside.

### 6.1 Direct AMPK Agonists

PT1, C24, and ZLN024 are known to be direct agonists of the AMPKα, which activate AMPK by inducing conformational changes that release autoinhibition without altering the intracellular AMP:ATP ratio. PT1 is an AMPKα1 activator that is independent of the upstream kinase LKB1, which activates AMPKα1 by interacting with residues Glu96 and Lys156 in the α1 subunit to separate the AID from the KD [68]. In an I/R-induced myocardial injury model in mice, PT1 promoted autophagy, enhanced cardiomyocyte survival by activating AMPKα*,* and exerted cardioprotective effects [69]. However, the low bioavailability of PT1 and its modulatory effects on mitochondrial respiration suggest that further structural optimization is required. C24 is a structurally modified product of PT1 with higher potency and bioavailability, and although it has a clear role in hepatic metabolism, studies on its cardiac-related effects are limited [70]. ZLN024, as a novel type of AMPK allosteric activator, not only activates AMPKα1 and α2, but also protects the Thr172 locus against the dephosphorylation of protein phosphatase 2c alpha (PP2Cα); however, studies in the heart are also limited [71]. Overall, direct α-subunit agonists have potential in treating HF, but their clinical applications remain limited by ongoing early-stage studies.

Direct agonists acting at the ADaM site include thienopyridines, pyrrolopyrimidines, benzimidazoles, indole acids, and tetrahydroquinoline, which act mainly on the AMPK complex containing the β1 subunit. A-769662 is a typical thienopyridine agonist, which activates AMPK via allosteric activation and inhibits PP2Cα-mediated Thr172 dephosphorylation [72]. In the heart, A-769662 selectively activates the α2/β1 subunit complex and synergistically enhances glucose uptake and attenuates oxidative damage when coupled with AMP-dependent agonists, but this synergistic effect is not associated with increased AMP concentrations [73]. In the AMPKα2 knockout mouse model, A-769662 still compensates for the function by activating AMPKα1 to promote myocardial contraction [74]. A-769662 also shows promising protective effects in the ischemic heart by preserving energy, delaying myocardial contracture, and reducing cell death [75]. However, A-769662 suffers from AMPK nondependent targeting activity, which may lead to adverse effects, thus limiting its potential for drug development.

Meanwhile, 5-aminoimidazole-4-carboxamide ribonucleoside (AICAr) is a widely used direct agonist of the γ-subunit that enters the cell via the adenosine transporter and generates ZMP, an AMP analog that mimics AMP binding to the adenylate-binding site, thereby activating AMPK [76]. AICAr primarily increases the activity of AMPKα2. Moreover, AICAr regulates energy metabolism in skeletal muscle cells by activating AMPKα2, with less effect on AMPKα1 [77]. Activation of AMPKα2 in the myocardium improves cardiac function and inhibits ferroptosis [78]. AICAr can exert protective effects during HF through multiple pathways. Indeed, in a rapidly paced canine model, AICAr attenuated apoptosis by increasing nitric oxide levels through AMPK activation, improving insulin resistance, and inhibiting fibrosis [79]. In a mouse model of HF, AICAr dynamically and bidirectionally regulated the myocardial autophagy homeostasis by activating the mTORC2-mediated phosphorylation of protein kinase B (AKT) at Ser473 to attenuate over-autophagy, while simultaneously restoring autophagy levels by inhibiting mTORC1 activity and preventing the phosphorylation of downstream effector molecules [80, 81]. However, AICAr is not a specific AMPK agonist, and its metabolite ZMP inhibits mitochondrial oxidative phosphorylation and affects other AMP-regulating enzymes through an AMPK-independent mechanism [82]. Additionally, AICAr, although exhibiting a short half-life, promotes side effects such as hypoglycemia and bradycardia. Thus, AICAr also does not represent a suitable candidate for treating HF, meaning more specific and less toxic AMPK agonists must be developed.

### 6.2 Indirect AMPK Agonists

Metformin is the first-line therapeutic agent for type 2 diabetes. Metformin inhibits hepatic glucose production by inhibiting mitochondrial complex I, elevating the intracellular AMP:ATP ratio, and indirectly activating AMPK. Recently, the cardioprotective effects of metformin in HF have received increased attention. The mechanisms of action of metformin include improving energy metabolism, maintaining mitochondrial homeostasis, inhibiting oxidative stress, and suppressing cell death. These effects are dose- and duration-dependent and are not exclusively dependent on AMPK. In a mouse model of HF, low-dose metformin (125 µg-kg-^1^-d-^1^) activated endothelial nitric oxide synthase (eNOS) and PGC-1α through an AMPKα2-dependent pathway, improving mitochondrial respiration and ATP synthesis, thereby protecting the heart. Comparatively, high doses of metformin (100 mg-kg-1-d-1) inhibited mTOR activation via the AMPKα2-nondependent pathway and alleviated HF [24, 83]. Alternatively, in a mouse model of myocardial I/R injury, metformin promoted cytoplasmic AMPKα1 and intranuclear AMPKα2 signaling, thereby improving autophagy flux and restoring cardiac function [84]. Overall, metformin modulates both AMPKα1 and AMPKα2 activities, and its agonistic effects are affected by dose and duration of action.

SGLT2 inhibitors are a new class of oral hypoglycemic drugs that reduce glucose reabsorption and lower blood glucose levels by inhibiting SGLT2 in renal tubules. Although SGLT2 is not expressed in the heart, the cardiac off-target effect of SGLT2 inhibitors offers potential for treating HF through a mechanism that may involve the activation of AMPK, which regulates energy metabolism, inflammatory responses, oxidative stress, and autophagy. Studies have found that canagliflozin, empagliflozin, and dapagliflozin activate AMPK in cardiomyocytes. The activation mechanism of AMPK by canagliflozin is similar to that of metformin, which activates AMPK by inhibiting mitochondrial complex I activity and increasing the intracellular AMP:ATP ratio [85]. Simultaneously, canagliflozin also has a strong inhibitory effect on SGLT1 and improves the redox state of cardiomyocytes by inhibiting AMPKα2 through suppression of SGLT1 expression, inhibiting the activity of NOX1/2, and enhancing the coupling of eNOS [86]. Alternatively, the specific mechanism of AMPK activation by empagliflozin and dapagliflozin has yet to be fully clarified; nonetheless, they exhibit significant HF protection potential [87]. The specific mechanism through which SGLT2 inhibitors act in HF has yet to be fully defined. Future studies must clarify the differences between various drugs, their potential side effects, and their clinical applications.

Resveratrol (RES) is a natural polyphenolic compound that has been shown to slow the progression of HF. Studies have shown that RES activates AMPK by inhibiting mitochondrial ATP synthase and phosphodiesterases (PDEs) [88]. In a mouse model of post-infarction HF, RES inhibited the mTOR/p70 ribosomal S6 kinase pathway, enhanced autophagy levels, attenuated myocardial hypertrophy, and improved cardiac function [89]. However, some studies have also shown that RES restored myocardial ATP levels and reduced AMPK activity, inhibiting excessive autophagy and improving cardiac function [90]. These differences may stem from the fact that RES has multiple pharmacological activities and can interact with different molecular targets to regulate AMPK activity and maintain autophagic homeostasis bidirectionally. Additionally, RES exerts cardioprotective effects through AMPK-independent mechanisms. As a SIRT1 agonist, RES reduces the acetylation level of p53 K382, decreases the degradation of solute carrier family 7 member 11, and increases the levels of glutathione and glutathione peroxidase 4 by activating SIRT1 in cardiomyocytes, thus reducing cardiomyocyte iron death and improving cardiac function [91]. Future studies should clarify the AMPK-related and unrelated mechanisms of RES-mediated cardioprotection further to optimize its application in HF therapy.

## 7. Conclusion

This article reviews the changes in many physiological processes after HF and their interaction with AMPKα. Indeed, AMPKα, the active AMPK subunit, is activated upon phosphorylation at the Thr172 site, which is important for improving HF. The article describes the general structure of AMPK and its relationship to the activation state, highlighting the key role of AMPKα in AMPK activation. The AMPKα subunit comprises two isoforms, AMPKα1 and AMPKα2, which differ significantly in tissue distribution, cellular localization, and roles in the heart. LKB1, as the main AMPK kinase, is activated by direct phosphorylation of Thr172. Phosphorylation of the Thr172 site activates AMPKα, while AMPKα2 activation largely depends on LKB1 phosphorylation. Activated AMPKα shows significant therapeutic potential in various cellular physiological processes in HF. In particular, the protective effects of AMPKα2, which is highly expressed in the heart, in HF include promoting the remodeling of energy metabolism, improving mitochondrial dysfunction, activating mitochondrial autophagy, attenuating oxidative stress, and reducing cardiomyocyte death. Therefore, targeted therapy against AMPKα2 may achieve better results in treating HF. Currently, AMPK agonists fall into two categories: direct and indirect. Although direct agonists have some α2 subtype selectivity, their selective activation ability is limited, and given the safety, efficacy, and pharmacokinetics of the drugs, there are no small-molecule direct AMPK agonists in clinical use, and only a few drugs have entered the clinical trial phase. Although indirect agonists are widely used in the clinic, these compounds depend on the presence of proteins upstream in the AMPK signaling pathway and have some effects independent of AMPK activation, with fewer factors controlled. Therefore, direct activators should be used as the main focus of AMPK-activating drug development to develop drugs that activate the AMPKα2 subtype with specific indications, which may contribute to the future prevention and treatment of HF.
